# Bayesian inference of population expansions in domestic bovines

**DOI:** 10.1098/rsbl.2007.0146

**Published:** 2007-05-29

**Authors:** E.K Finlay, C Gaillard, S.M.F Vahidi, S.Z Mirhoseini, H Jianlin, X.B Qi, M.A.A El-Barody, J.F Baird, B.C Healy, D.G Bradley

**Affiliations:** 1 Smurfit Institute of Genetics, Trinity CollegeDublin 2, Ireland; 2 Institute of Genetics, University of Berne3001 Berne, Switzerland; 3 Agricultural Biotechnology Research Institute of Iran (ABRII), 31535-1897 KarajIran; 4 Department of Animal Science, Faculty of Agriculture, Guilan University3179 Rasht, Iran; 5 Institute of Animal Science, Chinese Academy of Agricultural SciencesBeijing 100094, China; 6 Kunming Institute of Zoology, Chinese Academy of SciencesKunming, Yunnan 650223, China; 7 Animal Production Department Faculty of Agriculture, Minia UniversityMinia, Egypt; 8 Cardiff Centre for Lifelong Learning, Cardiff CF10 3XQUK; 9 Cambridge Institute for Medical Research, University of CambridgeCambridge CB2 0XY, UK

**Keywords:** domestication, expansion, demographic history, bovines

## Abstract

The past population dynamics of four domestic and one wild species of bovine were estimated using Bayesian skyline plots, a coalescent Markov chain Monte Carlo method that does not require an assumed parametric model of demographic history. Four domestic species share a recent rapid population expansion not visible in the wild African buffalo (*Syncerus caffer*). The estimated timings of the expansions are consistent with the archaeological records of domestication.

## 1. Introduction

The change from a hunter–gatherer lifestyle to an agricultural one had great implications for both humans and partner species. Genetic evidence suggests that the histories of many species involved several domestication events, separated by both time and space (Bruford *et al*. 2003), but each incorporating a limited number of progenitors. As most domestic species now number millions worldwide, their population histories must involve rapid expansions since domestication.

Mitochondrial DNA (mtDNA) is widely used in the study of domestication; its rapid mutation rate allows the accumulation of variation within the relevant time frame, and its maternal inheritance and lack of recombination mean that sequences can enter the population only by the domestication of a female animal.

Genetic signatures of domestication are read within mtDNA phylogenies by the identification of star-like patterns, suggesting expansion since the time of domestication (MacHugh & Bradley 2001). However, identification of such patterns is subjective and unclear for some species. While population expansion may also be inferred from mismatch histograms and such statistics as Tajima's *D* and Fu's *F*_s_ (Tajima 1989; Fu 1997), these may be difficult to relate to a particular time depth. Clear expansion signals are difficult to discern in complex phylogenies. While population expansions could have many causes, a large expansion since the time of domestication is presumed to be related to its effects.

Bayesian skylines are a recently developed methodology to infer past changes in population size from current genetic diversity within the population. Under coalescent theory, the probability of two lineages coalescing during a generation is inversely proportional to population size at that time. Skyline plots (Pybus *et al*. 2000) estimate effective population sizes during the intervals between coalescence events based on interval lengths. Bayesian skylines (Drummond *et al*. 2005) use Markov chain Monte Carlo sampling to create many thousands of genealogies and estimate the posterior distribution of population size at various time points. The mean, median and 95% highest posterior density (HPD) of the posterior distributions can be plotted over time; displaying a measure of uncertainty comprising both coalescent and phylogenetic errors. In contrast to many methods of estimating demographic history, no prior assumptions about population history or what constitutes a domestication cluster are required. Here, we analyse five mtDNA sequence data collections from within the tribe *Bovini* and show evidence for recent population expansion in four domestic species, but not in wild African buffalo.

## 2. Material and methods

All species were studied using mtDNA D-loop sequences: 71 yak (*Bos grunniens*) sequences of 284 bp in length; 110 water buffalo (*Bubalus bubalis*) of 842 bp; 24 mithan (*Bos frontalis*) of 414 bp; 195 African buffalo (*Syncerus caffer*) of 342 bp; and 84 cattle (*Bos taurus*) of 894 bp. Forty-one of the yak sequences, 34 of the water buffalo and all of the mithan were newly sequenced, all other data were publicly available. Neighbour-joining trees were drawn using the program ClustalX v. 1.81 (Thompson *et al*. 1997). Bayesian skyline plots were drawn using BEAST v. 1.2 (Drummond & Rambaut 2003). Accession numbers, sequencing details and details of the model used in BEAST are included in the electronic supplementary material.

As accurate mutation rates are not known for all the species in this study, a uniform rate of 32% Myr^−1^ has been applied. Although mutation rates can vary widely between species, all Bovini belong to the same tribe and diverged very recently. Shapiro *et al*. (2004) used Bayesian coalescent analysis of radiocarbon-dated bison D-loop sequences to estimate this rate. It agrees moderately well with the rates of 38 and 30.1% Myr^−1^ estimated from the hypervariable region and the complete D-loops of cattle (Bradley *et al*. 1996; Troy *et al*. 2001).

## 3. Results

Mitochondrial sequence neighbour-joining phylogenies generated from each sample are shown in figure 1. Previously described, phylogenetically distinct, clusters of sequences separated by long internal branches are visible in cattle and water buffalo (Bradley *et al*. 1996; Kierstein *et al*. 2004). These clusters represent separate domestication events, but it is difficult to draw conclusions from the diversity within clusters; phylogeographic structure may point towards further complexity in the domestication process (Troy *et al*. 2001). The yak and mithan phylogenies also contain long internal branches, suggesting multiple incorporations of divergent haplotypes into the domestic pool. However, in these species, it is more difficult to discern domestication expansion signals within the separated clusters and to define the limits of clusters. Indeed, these domestic phylogenies are not dramatically different from the wild African buffalo phylogeny.

In contrast, the skyline diagrams, which summarize instantaneous estimates of effective population size, show recent rapid population expansions for each of the domestic species within the last 10 000 years, the period since domestication (figure 2). However, the skyline plot for African buffalo implies a contrasting gradual population expansion, followed by a sharp decline.

## 4. Discussion

All the domesticated bovid samples examined here suggest a domestication signal: a recent, steep, continuous population expansion, post-dating the postulated time of domestication and attributable to the growth in domestic animal numbers linked to the spread and progression of agricultural practices.

Archaeological and genetic data suggest that cattle were domesticated in the Fertile Crescent, Southern Asia and Africa 10 000 years before present (YBP); yak in the Himalayas and Qinghai–Tibetan plateau 4500 YBP; water buffalo in the Indus valley 5000 YBP and/or in China as early as 7000 YBP; and mithan in the Indus valley 4500 YBP (Simoons 1968; Qian 1979; Cockrill 1981; Loftus *et al*. 1994; Troy *et al*. 2001; Hanotte *et al*. 2002; Wiener *et al*. 2003). Although the dating of events must be approached with caution, all expansions appear to have started at or after the time of domestication suggested by archaeological evidence.

While the order of expansion of the median values of the population size estimates appears to reflect the order in which the species were domesticated, the Bayesian 95% HPDs (figure 2*b*) of the different populations overlap, and inference of expansion event order is insecure. The application of a single mutation rate to all species facilitates the direct comparison of skyline diagrams and the relative timings of expansion events. The 95% HPD is influenced by several factors, including biases in rates, difficulty of calibration and violations of the assumptions of the coalescent. Several populations were combined in all analyses to maximize sample size. This also has unknown effects on the accuracy of estimation, but a skyline drawn only using European *Bos taurus* does not differ significantly in the shape or timing of the expansion from the skyline drawn using all cattle (results not shown). Uncertainty about the population size increases as time approaches the present. Jeffrey's prior was used to prevent artificial inflation of population sizes at the present. The expansion visible in the mithan median values may simply be due to the widening of the HPD towards the present. The mithan sample was the smallest and may not contain enough information to identify a significant population expansion signal. Such a signal is present in the other domestic datasets, in which both the upper and lower limits of the 95% HPD increase towards the present.

The shape of the African buffalo skyline diagram conforms to its known history. Epidemics and habitat destruction since the late 1800s have caused a decline in buffalo population size (Simonsen *et al*. 1998). It is possible that the population also declined for several 1000 years before this or that the reduction in genetic variety caused by the extreme bottleneck gave similar results to a more gradual population decline over a longer period. Previous genetic indications of a population expansion starting between 37 000 and 270 000 YBP agree with fossil record indications of a late Pleistocene origin and expansion of buffalo (Van Hooft *et al*. 2002) The buffalo skyline was drawn under the same model and using a dataset comparable to the domesticated animals. Its very different skyline shape, conforming to its known history, indicates that the expansions seen in the domesticated populations are not an artefact of the model or method. The buffalo skyline is different from that of another wild bovid, American bison. *Bison bison* showed a complex history of growth and decline, the population reached a maximum 30 000–45 000 YBP, declined until a severe bottleneck approximately 10 000 YBP, showed a population recovery and recently underwent another decline (Shapiro *et al*. 2004; Drummond *et al*. 2005). The bison bottleneck could be due to several causes, including climate change and human activity. The buffalo do not appear to have undergone a similar crisis, possibly due to differences in geographical location or pattern of distribution. Pre-bottleneck demographic history was visible in the bison skyline because ancient sequences were used. As skylines can reveal only the demographic history of the sequences included in the analysis, a dataset with a most recent common ancestor that post-dates a population bottleneck will not contain any information about population size or diversity before that bottleneck. Examination of ancient sequences in the domestic species might reveal a more complex pre-domestication bottleneck demographic history than the apparent constant population size shown here.

The presence of a consistent signal of recent population expansion in four domesticates and its absence in a related wild species are strongly suggestive that it is an effect of domestication. However, it could also be the signature of an older population expansion, such as the effects of postglacial population recovery, subsequently incorporated into the domestic population. Such pre-domestic expansion may be visible in wild boars (*Sus scrofa*) (Fang & Andersson 2006) and extinct European wild oxen (Edwards *et al*. 2007). The similarity of signal within the four domestic populations and its absence in African buffalo challenge a hypothesis of expansion through natural causes. The wild progenitors of these species inhabited very different biomes throughout Eurasia rendering it unlikely that the emergence from glaciation could have had such similar effects in each. However, because bison have also experienced a population expansion within the last 10 000 years, without being domesticated, it is not possible to attribute the expansions visible in the domestic species solely to the effects of domestication. Rather, they are probably a combination of the effects of Holocene postglacial recovery followed within a short period by the remarkable success of animal husbandry.

## Figures and Tables

**Figure 1:**
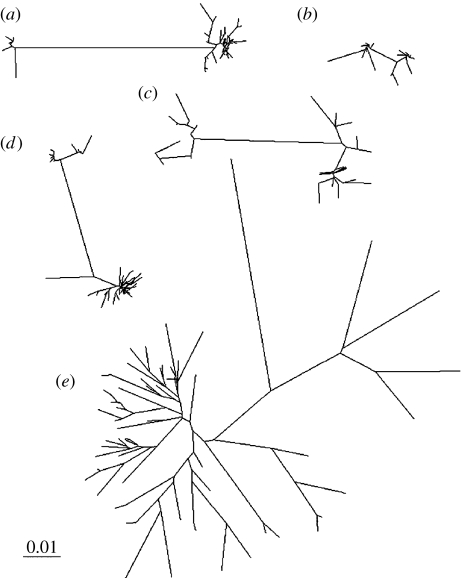
Neighbour-joining trees drawn using (*a*) 110 water buffalo, (*b*) 24 mithan, (*c*) 71 yak; (*d*) 84 cattle and (*e*) 195 African buffalo sequences. The scale bar indicates units of genetic distance. The cattle and water buffalo phylogenies show phylogenetically distinct clusters indicative of population expansions. The phylogenies of the yak, mithan and African buffalo samples are more complex and difficult to interpret.

**Figure 2:**
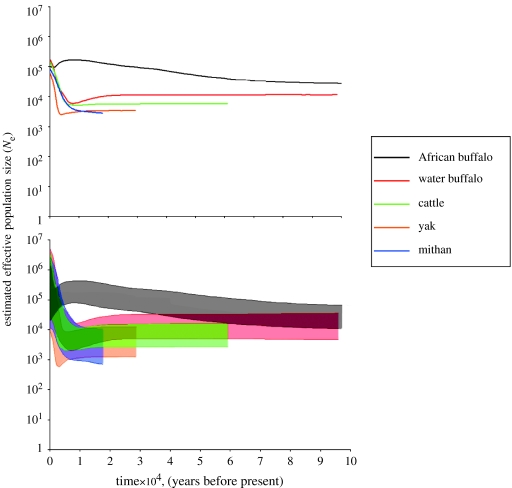
A comparison of (*a*) the median values and (*b*) 95% HPDs of the posterior distribution of *N*_e_ estimates for four domesticated and one wild bovine species. Note that only the HPDs should be considered for robust inference of change in population size. Time is plotted linearly; *N*_e_ is presented on a logarithmic scale. The HPDs of the domestic species show rapid recent population expansions since the time of domestication, while the African buffalo show a gradual population expansion followed by a decline.

## Supplementary Material

Sequence details Accession numbers of publicly available sequences used and sequencing details of newly generated data
