# Supplementary material for: Bayesian inference of population expansions in domestic bovines
Source: Biol Lett. 2007 May 29;3(4):449–52. doi: 10.1098/rsbl.2007.0146 (PMC2111054; doi:10.1098/rsbl.2007.0146)
Supplement: Supplementary file 1 [file rsbl20070146s28.doc]

# Sequences used in analysis

A total of 84 publicly available cattle sequences were used: AB085921to AB085923, AY337520 to AY337546, AY378133 to AY378137, AY521112 to AY521136, AY902382 to AY902405

94 publicly available African buffalo were used: AF028844, AF028849, AF028850, AF028855, AF028860, AF028862, AF028865, AF028874, AF028880, AF028882, AF028883, AF028885, AF028887, AF028888, AF028889, AF028893, AF028895, AF028898, AF028899, AF028903, AF028914, AF028915, AF028926, AF028928, AF028931, AF028933, AF028942, AF028947, AF028951, AF028952, AF028958, AF028959, AF028960, AF028964, AF028965, AF028971, AF028976, AF028977, AF028978, AF028983, AF028987, AF028996, AF029001, AF029005, AF029007, AF029010, AF029020, AF029027, AF029031, AF029034, AF029035, AF029036, AF029037, AF313151, AF313155, AF313156, AF313158, AF313164, AF313169, AF313171, AF313180, AF313181, AF313187, AF313190, AF313195, AF313201, AF313210, AF313212, AF313213, AF313220, AF313222, AF313232, AF313238, AF313241, AF313254, AF313262, AF313267, AF313276, AF313282, AF313283, AF313285, AF313289, AF313293, AF313298, AF313305, AF313309, AF313311, AF313315, AF313317, AF313322, AF313326, AF313331, AF313337, AF313341,

The newly sequenced water-buffalo consisted of 26 Iranian buffalo (11 Azarbayeja, 7 Khoozestani, 8 Mazandarani), 3 Egyptian and 5 Swamp buffalo from Thailand) (accession numbers DQ995682 to DQ995715)

The 76 publicly available water-buffalo sequences consist of AF197210, AF475184, AF475212, AF197197, AF197200, AF197203, AF197206, AF197208, AF197215, AF197216, AF197219, AF197221, AF197223, AF461794, AF462612, AF462614, AF462616, AF462618, AF462620, AF475160, AF475162, AF475164, AF475166, AF475170, AF475172, AF475174, AF475176, AF475178, AF475180, AF475182, AF475186, AF475188, AF475199, AF475201, AF475203, AF475204, AF475206, AF475208, AF475210, AF475214, AF475216, AF475218, AF475220, AF475223, AF475225, AF475227, AF475229, AF475231, AF475233, AF475235, AF475237, AF475239, AF475241, AF475243, AF475245, AF475247, AF475249, AF475251, AF475253, AF475255, AF475257, AF475259, AF475261, AF475263, AF475265, AF475267, AF475269, AF475271, AF475273, AF475275, AF475277, AY195589, AY195591, AY195594, AY195597, AY195599

A total of 41 yaks were newly sequenced, 24 from Switzerland, 6 from Nepal, 8 from Russia (accession numbers DQ995716 to DQ995756). The 30 publicly available yak sequences consist of AF083355, AF485066, AY374125, AY521137, AY521138, AY521139, AY521140, AY521141, AY521142, AY521143, AY521144, AY521145, AY521146, AY521147, AY521148, AY521149, AY521150, AY521151, AY521152, AY521153, AY521154, AY521155, AY521156, AY521157, AY521158, AY521159, AY521160, AY521161, AY722118, AY749414,

The 24 newly sequenced mithans come from Bhutan (accession numbers DQ995658

to DQ995681).

# Sequencing conditions

PCR and sequencing reactions were carried out with two overlapping pairs of primers, P27BF 5’-CTGGTCTTGTAAACCAGAGAA-3’, P27BR 5’-GAAAGAACCAGATGCCT-3’, and P28 F 5’-ATTACCATGCCGCGTGAA-3’, P28R 5’-TAACAGGAAGGCTGGGACCAA-3’, with annealing temperatures of 52°C and 60°C respectively.

PCR reactions were carried out in a DNA Engine Dyad Peltier Thermal Cycler and consisted of two minutes at 94°C, 35 cycles of 40 seconds at 94°C, 40 seconds at the annealing temperature, and 40 seconds at 72°C, followed by four minutes at 72°C. Each 50 μl reaction contained 5μl each of dNTPs (2mM of each dNTP), MgCl (15 to 25mM), 10X reaction buffer, and both primers (4mM), 0.5 μl of Platinum Taq (Invitrogen), 32.5μl of sterile ddH2O and 2μl of the DNA template. PCR products were cleaned using Jetquick PCR purification spin kits by Genomed and sequenced by MWG Biotech (www.mwg-biotech.com).

**Beast analsysis**

Beast v 1.2 (Drummond and Rambaut 2003) was used to create a Bayesian skyline plot with 10 steps. The analysis was run for 10^7^ iterations with a burn-in of 10^6^ under the HKY +Γ +I model. Genealogies and model parameters were sampled every 1000 iterations. All operators were automatically optimised. Jeffrey’s prior was invoked for the first population size and an upper limit to the population size was set as 1x10^9^. Convergence of the chains to the stationary distribution was confirmed by visual inspection of plotted posterior estimates. The effective sample size (the number of effectively independent draws from the posterior distribution to which the Markov chain is equivalent) for each parameter exceeded 100, usually by an order of magnitude.
